# Hemodialysis patients’ satisfaction with dialysis care: a cross-sectional prospective study conducted in a non-profitable care facility, Minia Egypt

**DOI:** 10.1186/s12882-022-03010-3

**Published:** 2022-12-06

**Authors:** Noha H. Helmy, Amal Hussein, Marwa Kamal, Osama El Minshawy, Engy A. Wahsh

**Affiliations:** 1 Department of clinical pharmacy, Faculty of Pharmacy, Minia University, 61519 Minia, Egypt; 2 Department of clinical pharmacy, Faculty of Pharmacy, Fayoum University, 63514 Fayoum, Egypt; 3 Department of Internal Medicine, school of medicine, Minia University, 61519 Minia, Egypt; 4 Department of clinical pharmacy, Faculty of Pharmacy, October 6 University, 12573 Giza, Egypt

**Keywords:** Hemodialysis, Patient satisfaction, Dialysis care

## Abstract

**Background:**

The prevalence of chronic kidney disease (CKD) and end-stage kidney disease (ESKD) is increasing continuously as a result of the dramatic growth in the prevalence of two main causes of ESKD which are diabetes mellitus (DM) and hypertension, hence, ESKD represents a global concern. Based on the sixth annual report of the Egyptian society of nephrology, the prevalence of ESKD in Egypt is estimated to be 375 per 1000,000. Meanwhile, other studies estimated the prevalence in El-Minia governorate to be around 308 per 1000,000. Hemodialysis (HD) represents the main modality of Kidney replacement therapy (KRT) for sufferers of ESKD in El-Minia governorate. Patients treated with in-center HD attend dialysis care usually three times per week for several hours each time, hence, their experiences during dialysis care will likely have a major impact on living with chronic illness. Hence, measuring patient satisfaction is very important as it is not only an outcome but also a contributor to other outcomes and objectives, it can provide valuable information about problem areas that can be modified to improve patient experience and outcomes.

**Methods:**

A single-center cross-sectional prospective study was conducted in the HD unit, Minia nephrology and urology university hospital. Demographic data were obtained through face-to-face interviews, Patients received a questionnaire to assess satisfaction with medical staff interactions, as well as care before, during, and after dialysis. An observational checklist of healthcare staff and equipment in the dialysis unit was also given to the patients.

**Results:**

One hundred nineteen patients participated in the study; patients were generally satisfied with the care provided in the dialysis unit (mean = 2.64), patients were most satisfied with aspects of care related to nurses, while they were neutral about aspects related to physicians, and were dissatisfied with nutritional care.

**Conclusion:**

There are multiple problem areas in the HD unit affecting patients’ experience, and further improvement in the care provided in the dialysis unit is required.

**Supplementary Information:**

The online version contains supplementary material available at 10.1186/s12882-022-03010-3.

## Introduction

All developed nations are concerned about the rising expense of health care, which requires better utilization of available resources [1]. Measuring healthcare efficiency became necessary to establish if resources were spent effectively [2]. Accordingly, patient satisfaction measures care efficiency. Moreover, consumers have transformed from passive to active. Today’s healthcare consumers are more knowledgeable and critical of the services they receive [3].

Satisfaction” is defined as “the fulfillment of one’s wishes, expectations, or needs” [4]. Patient satisfaction indicates healthcare services meet patients’ needs, desires, or expectations [5]. Patient satisfaction is multifaceted [6], Each person’s features, beliefs, values, perceptions, emotions, and health circumstances determine its meaning [7], in addition to Previous healthcare experiences and how a patient views “care” [8]. Hence, patient satisfaction does not have one simple definition agreed upon by all researchers [4].

Chronic kidney disease (CKD) is kidney damage or a glomerular filtration rate (GFR) of less than 60 mL/min/1.73m^2^ for no less than 3 months. CKD can eventually lead to end-stage Kidney disease (ESKD) which represents the last stage of CKD when kidney replacement therapy (KRT) becomes a must [9]. Globally, the estimated prevalence of CKD is 13.4% (11.7–15.1%), while patients with ESKD are estimated to measure up between 4.902 and 7.083 million [10]. In a study by Afifi and colleagues on leading causes of ESKD, they mentioned the prevalence of ESKD in Egypt to be 375 pmp, based on the sixth annual report of the Egyptian society of nephrology [11]. A cross sectional study conducted in El-Minya governorate estimated the prevalence of ESKD to be 308 pmp [12]. There are three modalities of KRTs available for ESKD patients: transplantation, HD, and peritoneal dialysis. Although transplantation is the best treatment as it improves patients’ quality of life and reduces the expenses, it is not the most common KRT [13, 14]. HD represents the main modality of KRT in El-Minia governorate [15]. Patients treated with in-center HD attend dialysis care usually three times per week for several hours each time, hence, their experiences during dialysis care will likely have a major impact on living with chronic illness [16–18].

The study of patient satisfaction provides information about problem areas of care and even the success and failure of the health-care organization [19]. Healthcare staff can use provided information to guide corrective interventions in the health-care system [20–22]. Therefore, the current study aims to assess patients’ satisfaction with care at HD unit, Minia university.

Tables 1, 2 and 3 show a summary of studies found in the literature measuring patient satisfaction in developed countries, developing countries, and Egypt. Showing only two studies in Egypt revealing a need for further investigation in Egyptian HD population.

**Table 1:** Summary of published studies on HD patient satisfaction in developed countries

Authors	Year	Aim of study	Number of patients	Results
[23]	1987	Assess patient satisfaction with care and the association between satisfaction and QoL.	416 HD patients	Patients were generally satisfied with care especially with aspects related to physicians, patients with lower education levels were more likely to be satisfied.
[24]	1997	Identify attributes of dialysis care and rank them according to their importance to dialysis patients.	86 dialysis patients	Issues related to nephrologists, other doctors, and nurses had the highest ranking among attributes to dialysis care according to patients’ perspectives.
[25]	2002	Assess satisfaction with dialysis care.	79 dialysis patients	Patients with low levels of satisfaction with nephrologists had lower attendance rate to dialysis treatment.
[26]	2007	Assess satisfaction with dialysis care.	758 dialysis patients	Notable association was found between inter-dialysis weight gain and risk of dissatisfaction.

**Table 2:** Summary of published studies on HD patient satisfaction in developing countries

Authors	year	Aim	Number of patients	Results
[27]	2010	Assess patient satisfaction and the overall effect of dialysis on life.	322 HD patients	Mean overall dialysis satisfaction was 7.41 ± 2.75
[28]	2013	Evaluate patient satisfaction towards nursing care.	150 HD patients	90.5% of patients were satisfied with the patient-nurse relationship, unemployed patients were found to be more satisfied than employed patients.
[29]	2013	Evaluate patient satisfaction towards nursing care.	-	67.8% of patients reported satisfaction with nursing care.
[30]	2014	Assess patient satisfaction with HD care.	2145 patients	Most patients reported excellent or very good care, older patients were more likely to rate care as excellent.
[31]	2021	Assess patient satisfaction with HD care.	141 patients	The majority of patients reported satisfaction with nursing care except for time spent with the doctor. Married, and employed patients with good income reported better QoL.

**Table 3:** Summary of published studies on HD patient satisfaction in Egypt

Authors	year	aim	Number of patients	Results
[32]	2015	Explore the opinion of HD patients about the dialysis unit.	69 patients	Patients were mostly satisfied with doctors’ performance and less satisfied with food services.
[33]	2016	Assess patient satisfaction with HD care.	79 patients	Patients were generally unsatisfied except for time spent with the doctor, accessibility, and convenience.

## Methods

### Study design and setting

A cross-sectional prospective single-centered study conducted in HD unit, Minia nephrology, and urology hospital. Patients were recruited from HD unit between July 2020 and February 2021. The study aims to assess patient satisfaction with the care provided in the HD unit.

This study was approved by “the commission on the ethics of scientific research”, faculty of pharmacy, Minia university with code number: HV09/2020. Researchers ensured complete confidentiality of any information obtained from the patients.

### Inclusion and exclusion criteria

#### Inclusion criteria

Patients who were < 18 and > 85 years old undergoing maintenance dialysis who are willing to participate.

#### Exclusion criteria

Prescence of any diagnosed mental disease or dementia.

### Data collection

#### Study instruments

Demographic data and dialysis characteristics including (age, marital status, residence, education, occupation, duration of disease, and duration of dialysis) were collected from patients.

The researcher used a structured questionnaire designed to evaluate patient satisfaction with all aspects of care provided in HD unit, the questionnaire consists of three domains: Patient satisfaction with medical staff-patient interaction in the hemodialysis unit, Patient satisfaction and perception of care during dialysis session as well as Patient satisfaction and perception of care before and after dialysis session.

Each domain contained multiple items (total: 16 items) to which patients answered as dissatisfied, neutral, satisfied, or very satisfied (Likert- 4-point scale) as mentioned in Table 4.

**Table 4:** Interpretation of a Likert − 4- point scale

Dissatisfied	Neutral	Satisfied	Very satisfied
1-1.74	1.75–2.49	2.5–3.24	3.25-4

Patients also responded to an observational checklist regarding health-care staff and equipment in HD unit.

#### Validity and reliability

The tool was developed and translated to Arabic language and examined by 3 experts in the field of internal medicine and nephrology (Minia university, Minia, Egypt), modifications to some items were made accordingly. The instrument showed reliability and internal consistency after Cronbach’s alpha calculation as shown in Tables 5 and 6.

**Table 5:** Correlation Coefficients between the scale items and the total questionnaire

Item	R	α*
1. Welcome to renal unit.	0.702	**0.910**
2. Nurses’ attitude.	0.628	0.852
3. Explanation for delays.	0.845	**0.915**
4. Explanation of nature of treatment.	0.833	**0.926**
5. Handling complaints.	0.727	0.872
6. Nurses’ monitoring of dialysis.	0.706	0.811
7. Response to medical hitch.	0.686	0.812
8. Medication administration.	0.667	0.849
9. Nurses’ enquiry to physicians	0.755	**0.931**
10. Response of enquired physicians	0.777	**0.922**
11. Catheter site dressing.	0.661	0.813
12. Physical exam before dialysis.	0.762	**0.937**
13. Nurses’ observations before dialysis.	0.359	0.765
14. History of previous dialysis.	0.673	**0.928**
15. Nurses’ observation post dialysis.	0.775	**0.910**
16. Medical staff counseling post dialysis.	0.830	**0.929**

α ≥ 0.9 = excellent, 0.9 > α ≥ 0.8 = very good, 0.8 > α ≥ 0.7 = acceptable

*Significant at 0.05 level

**Table 6:** Correlation Coefficients and Alpha between each domain and the total questionnaire

Domain	R	Alpha*	η^2^
I- patient satisfaction with medical staff-patient interaction in hemodialysis unit	0.817	0.956	0.994
II- patient satisfaction and perception of care during dialysis session	0.899	0.954	0.997
III- patient satisfaction and perception of care before and after dialysis session	0.840	0.997	0.988

*Significant at 0.05 level

A pilot study was conducted including 15 patients to assess the clarity and applicability of the developed tool, the 15 patients were also included in the final study subjects.

### Statistical analysis

Data entry and statistical analysis were done using SPSS statistical software package.

## Results

**Fig. 1:**
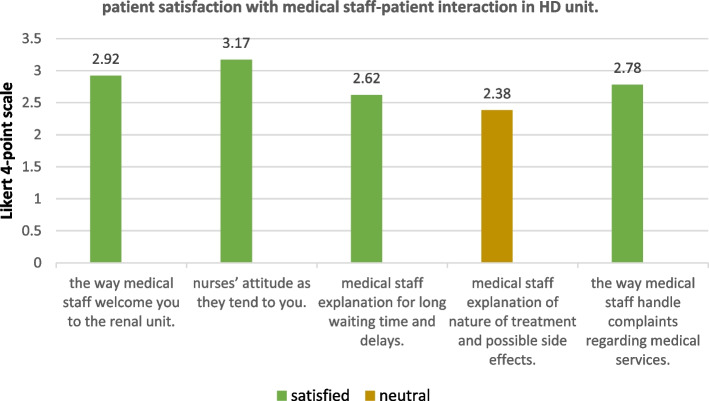
Patient satisfaction with medical staff-patient interaction in HD unit

**Fig. 2:**
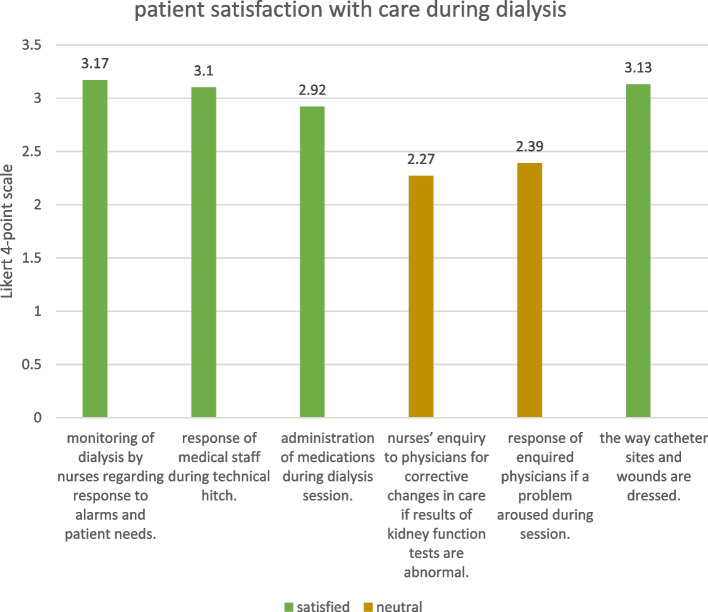
Patient satisfaction with care during dialysis

**Fig. 3:**
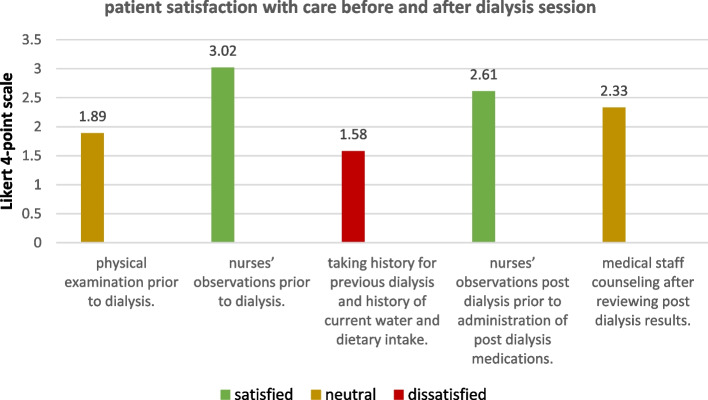
Patient satisfaction with care before and after dialysis session

All demographic data of the participants are detailed in Table 7. Patient satisfaction with medical staff- patient interaction in HD unit and patient satisfaction with care before, during, and after dialysis (*n* = 119) were expressed as percentages, as shown in Figs. 1, 2 and 3.

**Table 7:** demographics of study population

**Demographic data**		**N (%)**
**Sex**	Male	**42**(40.8%)
	Female	**61 **(59.2%)
**Marital status**	Married	**82 **(79.6%)
	Not married	**21**(20.4%)
**Residence**	Rural	**63 **(61.2%)
	Urban	**39 **(38.8%)
**Education**	Illiterate	**30 **(25.2%)
	Read and write	**33 **(27.7%)
	Primary	**4 **(3.4%)
	Secondary/technical	**25 **(21%)
	High	**27 **(27.7%)
**Occupation**	Housewife	**44** (37%)
	Not working	**49** (41.2%)
	Farmer	**5** (4.2%)
	Unskilled worker	**5** (4.2%)
	Skilled worker	**4 **(3.4%)
	Professional	**12** (10.1%)

A total of 119 patients participated in our study during the study period (The total number of patients in the HD unit is 160 patients, response rate is 74.3%). The mean age of the study sample was 47.5 years (range: 19:80), females were slightly more than males representing 56.3% of the study population, 79% of the study population were married ,63.9% were from rural areas.

Regarding patients’ response to the questionnaire, patients were generally satisfied with “medical staff-patient interaction in hemodialysis unit” (mean = 2.7), patients were satisfied with all aspects of this domain except for “medical staff explanation of nature of treatment and possible side effects” for which patients were neutral (mean = 2.38).

Independent samples t-test showed no significant difference between males and females in satisfaction with this domain (*p*-value = 0.870), also no significant difference in satisfaction was found between different marital status (*p*-value = 0.764), different residence (*p*-value = 0.271), different education levels (*p*-value = 0.202), or different occupation (*p*-value = 0.110), (significant at P ≤ 0.05).

A non-significant correlation was found between sex (*r*=-0.15, *p* = 0.870), marital status (*r*=-0.0.028, *p* = 0.764), residence (*r*=-0.102, *p* = 0.271), and occupation (*r*=-0.078, *p* = 0.4) and patient satisfaction with this domain. A significant negative poor correlation was found between education and patient satisfaction (*r*=-0.084, *p* = 0.03).

Patients were also generally satisfied with “care during dialysis session” (mean = 2.83), patients were satisfied with all aspects of this domain except for “nurses’ enquiry to physicians for corrective changes in care if results of kidney function tests are abnormal” and “response of enquired physicians if a problem aroused during session” for which patients were neutral (mean = 2.27 and 2.39 respectively).

Independent samples t-test showed no significant difference between males and females in satisfaction with this domain (*p*-value = 0.616), also no significant difference in satisfaction was found between different marital status (*p*-value = 0.729), different residence (*p*-value = 0.897), different education levels (*p*-value = 0.912), or different occupation (*p*-value = 0.340), (significant at *P* ≤ 0.05).

A non-significant correlation was found between sex (*r*=-0.046, *p* = 0.616), marital status (*r*=-0.032, *p* = 0.729), residence (*r*=-0.012, *p* = 0.897), occupation (*r*=-0.125, *p* = 0.177), education (*r*=-0.084, *p* = 0.367) and patient satisfaction with this domain.

Patients were generally neutral about “care before and after dialysis session” (mean = 2.28), Patients were satisfied with only two aspects of this domain; “nurses’ observations prior to dialysis” and “nurses’ observations post dialysis prior to administration of post dialysis medications”; (mean = 3.02 and 2.6 respectively), while Patients were neutral about two aspects of this domain; “physical examination prior to dialysis” and “medical staff counseling after reviewing post dialysis results”; (mean = 1.89 and 2.33 respectively), Patients were dissatisfied with “taking history of previous dialysis and history of current water and dietary intake”; (mean = 1.58).

Independent samples t-test showed no significant difference between males and females in satisfaction with this domain (*p*-value = 0.986), also no significant difference in satisfaction was found between different marital status (*p*-value = 0.072), different residence (*p*-value = 0.561), different education levels (*p*-value = 0.609), or different occupation (*p*-value = 0.190), (significant at *P* ≤ 0.05).

A non-significant correlation was found between sex (*r*=-0.002, *p* = 0.986), marital status (*r*=-0.165, *p* = 0.072), residence (*r*=-0.054, *p* = 0.561), occupation (*r*=-0.144, *p* = 0.119) and education (*r*=-0.113, *p* = 0.222) and patient satisfaction with this domain.

On calculating the mean of all 16 questions included in the questionnaire, it was found that Patients were generally satisfied with care they received at dialysis unit (mean = 2.64).

Thirty-eight patients attended the morning session (31.9% of total participants), their mean level of satisfaction was 2.63 on Likert 4-point scale, while 48 patients attended the afternoon session (40.3%), and their mean level of satisfaction was 2.72 on Likert 4-point scale. 33 patients attended the evening session (27.7%) and their mean level of satisfaction was 2.61 on Likert 4-point scale). Pearson’s correlation coefficient was calculated to assess the correlation between session timing and level of satisfaction: *r* = 0.018, *P*-value = 0.842, showing that there is no significant correlation between session timing and level of satisfaction

On analyzing patients’ responses to the observational checklist regarding health-care staff, the majority of the patients (77.3%) reported a deficiency in nephrologists in the hemodialysis unit, on the contrary, the majority (79%) were satisfied with nurse-patient ratio. 72.3% and 66.4% were satisfied with the availability of biomedical technologists and lab technologists respectively. However, 88.2% of the patients were not satisfied with the availability of nutritionists for dietary counseling. Almost all the patients (96.6%) were satisfied with the supportive staff and cleanup process.

The observational checklist regarding equipment in HD unit showed that the majority of patients (62.2%) reported that available dialysis machines are not enough, while (58.8%) reported that dysfunctional dialysis machines are repaired in time. All patients (100%) reported that miscellaneous items are always available to facilitate dialysis, and that they don’t need an item store for dialysis items.

## Discussion

In the past, health care providers assumed that they knew patients’ needs based on professional standards and their assessment [34], in the present, due to the increasingly competitive health-care environment, and continuously increasing patient awareness, health-care providers bear more attention to patients’ satisfaction with health-care [35]. Moreover, consumers’ attitude has dramatically changed, moving from a passive role to an active one. Nowadays, users of health-care services are better informed, hence, they are more critical towards the services provided to them [3]. The study of patient satisfaction provides information about problem areas of care and even the success and failure of the health-care organization [19], Healthcare providers can use provided information to guide corrective interventions in the health-care system [20–22].

In the current study, it was found that patients were more satisfied with aspects of care related to nurses than physicians; as 79% of the patients were satisfied with the nurse-patient ratio at the HD unit, while 77.3% reported a deficiency in the nephrologists. In addition, patients were neutral about nurses’ enquiry to physicians for corrective changes in care if results of kidney function tests are abnormal, as well as the response of the enquired physicians if a problem aroused during dialysis (mean = 2.27, 2.39 respectively), on the other hand, patients reported satisfaction with nurses’ attitude and monitoring of dialysis, as well as the catheterization techniques and dealing with wounds (mean = 3.17, 3.17, 3.13 respectively) which are aspects of care related to nurses. Nutritional care also represented a problem area, as patients were dissatisfied with taking history of water and dietary intake (mean = 1.58), while 88.2% of patients reported scarcity of nutritionists available for dietary counseling.

The study conducted by Rubin and colleagues in 1997 showed that issues related to nephrologists had the highest ranking among attributes to dialysis care [24], while Kovac and colleagues discovered that lower levels of satisfaction with nephrologists led to lower attendance rates [25], emphasizing the great need to increase patients’ satisfaction with nephrologists in the dialysis unit to improve quality of life and patient outcomes.

The results of the current study coincided with the results of a study by Mansour et al. that reported high satisfaction regarding nursing care and communication between patients and nurses (86.5% and 90.4% respectively) [28]. On the contrary, a study by Ferrans et al. found that patients were most satisfied with aspects of care related to physicians, followed by aspects related to nursing/dialysis treatment [23].

In agreement with the current study, a study conducted in Kenyatta national hospital Nairobi, Kenya, found that patients were generally satisfied with nursing services (67.8%), but the main cause of dissatisfaction was the inappropriate nurse-patient ratio, which didn’t represent a problem for the patients of the current study as 79% were satisfied with nurse-patient ratio, another cause of dissatisfaction in the mentioned study was inadequate number of dialysis machines, same as the current study as 62.2% of patients reported that dialysis machines are not enough [29]. Another study conducted in HD unit of Lahore general hospital, Pakistan, found that the majority of patients (82.56%) were satisfied with care they receive at the dialysis unit, except for time spent with doctor, supporting the results of the current study [31].

In Egypt, two studies were found in the literature measuring patient satisfaction, a study conducted in Beni-suef university hospital found that patients were generally unsatisfied except for time spent with doctor (64.6%), the other study conducted in Mansoura, Egypt, found that the highest level of satisfaction was for doctors’ performance (85.5%), both studies contraindicating the findings of the current study as patients were not highly satisfied with aspects related to physicians [32, 33].

The study results were discussed with the administrative board in charge of the HD unit, the researchers advised increasing the number of residents in the HD unit, as well as implicating a patient education program, and also adding a clinical pharmacist and a nutritionist to the dialysis care team; to help solve problem areas and increase patient satisfaction.

## Conclusion

Patients were generally satisfied with care provided at the dialysis unit, the findings of the current study uncovered some problem areas related to availability of physicians and nutritionists, as well as the inadequate number of dialysis machines. Further improvement and modifications are required to increase patient satisfaction.

## Supplementary Information

**Additional file 1.**

## Data Availability

Data available on request from the corresponding author.

## Abbreviations

CKD: Chronic kidney disease
ESKD: End-stage kidney disease
DM: Diabetes mellitus
HD: Hemodialysis
KRT: Kidney replacement therapy
