# Supplementary material for: Hemodialysis patients’ satisfaction with dialysis care: a cross-sectional prospective study conducted in a non-profitable care facility, Minia Egypt
Source: BMC Nephrol. 2022 Dec 6;23:387. doi: 10.1186/s12882-022-03010-3 (PMC9724252; doi:10.1186/s12882-022-03010-3)
Supplement: Supplementary file 1 — Additional file 1. [file 12882_2022_3010_MOESM1_ESM.pdf]

Name:	Sex:	Age:
Marital status: married O not married O		
Education: illiterate O read and write O primary O secondary/technical O high O		
Occupation: housewife O not working O farmer O unskilled worker O skilled worker O professional O		
Residence: rural O urban O		
Duration of disease:	Duration of dialysis:	

I- patient satisfaction with medical staff-patient interaction in hemodialysis unit:

Items	dissatisfied	neutral	satisfied	v. satisfied
1. The way medical staff welcome you to the renal unit.				
2. Nurses' attitude as they tend to you.				
3. Medical staff explanation for long waiting time and delays.				
4. Medical staff explanation of nature of treatment and possible side effects.				
5. The way medical staff handle complaints regarding medical services.				

II- patient satisfaction and perception of care during dialysis session:

Items	dissatisfied	neutral	satisfied	v. satisfied
1. Monitoring of dialysis by nurses regarding response to alarms and patient needs.				
2. Response of medical staff during technical hitch.				
3. Administration of medications during dialysis session.				
4. Nurses' enquiry to physicians for corrective changes in care if results of kidney function tests are abnormal.				
5. Response of enquired physicians if a problem aroused during session.				
6. The way catheter sites and wounds are dressed.				

III- patient satisfaction and perception of care before and after dialysis session:

Items	dissatisfied	neutral	satisfied	V. satisfied
1. Physical examination prior to dialysis.				
2. Nurses' observations prior to dialysis.				
3. Taking history for previous dialysis and history of current water and dietary intake.				
4. Nurses' observations post dialysis prior to administration of post dialysis medications.				
5. Medical staff counseling after reviewing post dialysis results.				

Observation checklist from patient perspective:

(A) Regarding health-care staff:

Questions	yes	no
1. Are there enough nephrologists available for treatment and prescription?		
2. Is nurse-patient ratio adequate?		
3. Are there enough biomedical technologists for repair and disinfection of dialysis machines?		
4. Are lab technologists available to facilitate kidney function tests and other relevant investigation?		
5. Are there nutritionists available for dietary counseling?		
6. Are there supportive staff to aid in environmental cleanup?		

(B) regarding equipment:

questions	yes	no
1. Are there enough dialysis machines?		
2. Are the dysfunctional dialysis machines repaired in time?		
3. Are there enough dialysis items in the item store(blood lines – bicarb. Solution) ?		
4. Are miscellaneous items always available to facilitate dialysis(dressing packs – gauzes – disinfectant lotions)?		
